# Drivers and Facilitators of HIV-Related Stigma in Healthcare Settings in Ireland

**DOI:** 10.1007/s10461-024-04489-7

**Published:** 2024-09-13

**Authors:** Elena Vaughan, András Költő

**Affiliations:** The Health Promotion Research Centre, School of Health Sciences, College of Medicine, Nursing and Health Sciences, University of Galway, University Road, Galway, H91TK33 Ireland

**Keywords:** HIV stigma, Healthcare settings, Healthcare workers, U = U, Europe, Ireland

## Abstract

People living with HIV who experience stigma in healthcare settings are at increased risk for engaging in health avoidance behaviours, suboptimal adherence to antiretroviral therapy, and viral non-suppression. HIV-related stigma erodes trust between patients and healthcare providers, thereby undermining both individual and public health. This study aimed to identify predictors of stigmatising attitudes, stigma practices, and fear of occupational transmission among healthcare workers in the Republic of Ireland. Data were collected from 295 healthcare workers using a standardised tool designed to measure HIV-related stigma. The outcomes examined were stigmatising attitudes, stigmatising practices (such as excessive infection precaution measures), and fear of occupational transmission. Multiple linear regression analyses were conducted to explore predictors at the individual, clinic, and policy levels. The results indicated that none of the models significantly predicted stigmatising attitudes. However, stigmatising practices were positively associated with never having worked in an HIV clinic, lack of knowledge or agreement with the concept of ‘undetectable equals untransmittable’ (U = U), and the presence of institutional policies, collectively accounting for 25.3% of the variance. Fear of occupational transmission was positively predicted by gender and lack of knowledge or agreement with U = U, explaining 23.8% of the variance. The findings highlight the critical role of U = U knowledge in reducing stigma-related behaviours and fears among healthcare workers. Enhancing knowledge and acceptance of U = U as part of comprehensive stigma interventions may help reduce the stigma experienced by people living with HIV in healthcare settings.

## Resumen

Las personas que viven con el VIH que se enfrentan con el estigma en el contexto de cuidado médico tienen un riesgo elevado de evitar la asistencia médica, de no cumplir con el tratamiento, y de sufrir fracasos de tratamiento. El estigma relacionado con el VIH hace daño a la confianza entre los profesionales de salud y los pacientes, y tiene efectos negativos por la salud individual y la salud pública. Comprender a los factores que promueven el estigma en el contexto de cuidado médico es fundamental para que se puedan crear soluciones para abordar el problema. Esta investigación tuvo como objetivo identificar a los predictores del miedo de transmisión del VIH entre los profesionales de salud en su sitio de trabajo, y de las actitudes y las prácticas estigmatizantes entre ellos. Hemos analizado datos que se recogieron de 295 profesionales de salud en Irlanda utilizando un cuestionario validado. Los resultados muestran que los predictores de las prácticas estigmatizantes incluyen no haber trabajado en una clínica de VIH; no tener conocimiento del U = U; y la presencia de políticas institucionales. El género y la falta de conocimiento del U = U eran predictores del miedo de transmisión del VIH. Mejorando el conocimiento del U = U entre los profesionales de salud puede ser una estrategia efectiva para que se reduzca el estigma en los contextos de cuidado médico.

## Introduction

Enormous progress has been made in the treatment of HIV in recent years. People living with HIV (PLHIV) now have lifespans comparable to HIV negative individuals [1], and do not transmit the virus once the treatment goal of viral undetectability has been reached [2]. Despite these advances, HIV-related stigma continues to affect the health and well-being of PLHIV and remains a barrier to stemming the epidemic [3]. The effects of stigma experienced in healthcare settings are well-documented [4]. Experiences of stigma in healthcare settings erode trust in healthcare providers [5, 6], and are associated with health avoidance behaviours including delays in seeking care [7] and delaying or avoiding appointments [8], which can negatively affect engagement and retention in care [4, 6]. Recent studies show that stigma experienced or perceived in healthcare settings is associated with adverse mental health impacts, including depressive symptoms and avoidance coping [6]. A number of studies have found that HIV stigma was associated with reduced or sub-optimal adherence to treatment [9–11], while stigma experiences in healthcare settings have also been shown to predict viral non-suppression [6, 9]. The latter can result in negative HIV-related health outcomes on an individual level, but may additionally have adverse implications for public health given the efficacy of treatment as prevention (TasP) [12]. Consequently, understanding and addressing HIV-related stigma in healthcare settings is vital to progressing global goals of eliminating the HIV epidemic [13].

In line with the socio-ecological perspective [14], HIV-related stigma generally is understood to encompass a variety of interactions and experiences at the individual and interpersonal level, the organisational/environmental level, and the broader structural level [15]. Similarly, HIV-related stigma in healthcare settings is conceptualised through a socio-ecological lens as occurring at different levels of social interaction [16–20]. The Health Stigma and Discrimination Framework is exemplary of the socio-ecological approach [15]. The framework identifies factors that trigger and reproduce stigmatising behaviours in healthcare settings [15]. At the individual level stigma ‘drivers’ encompass fears, negative attitudes, beliefs and stereotypes, and a lack of knowledge about HIV and/or the stigma attached to it. In order to differentiate from individual level aspects, the term ‘facilitators’ is used to capture structural and institutional level factors such as occupational practices, institutional-level policies (or lack thereof), laws, and gender and culture norms.

Stigma drivers and facilitators in healthcare settings can combine and interact to be promotive of stigmatising behaviours and attitudes towards PLHIV, which may range from outright denial of care, reduced quality or altered conditions of service, use of stigmatising language or inappropriate questions, and other stigmatising behaviours, such as the use of excessive infection precaution measures [7, 8, 10]. As well as acting as a descriptive model, the Health Stigma and Discrimination framework is a useful analytical tool. It is applicable in a range of contexts, and can help to identify intersections between stigma drivers and facilitators, as well as apposite domains for the targeting of interventions specific to culture and context. For instance, previous studies have used it to conduct exploratory research on healthcare worker (HCW) perspectives in Indonesia [21], and to carry out formative research to inform development of a HIV training programme in Ghana [22]. In the Ghanian study, cultural attitudes and beliefs about men who have sex with men (MSM), and a lack of awareness of how these unconsciously manifest during service delivery, were identified as key drivers of stigma in that context [22]. In the Indonesian study it was reported that gaps in knowledge about HIV transmission were implicated in healthcare worker fears of occupational transmission, pointing to a need for better HIV education [21]. Similarly, this study sought to identify what factors underpin key drivers and facilitators of HIV stigma among healthcare workers in the Republic of Ireland, in order to inform recommendations for intervention and future research.

## Aims

The present study was part of a broader mixed-methods project exploring HIV-related stigma in healthcare settings in the Republic of Ireland [23]. The project involved two national surveys – one of PLHIV and another of healthcare workers – and interviews (*n* = 16) with stakeholders including PLHIV, service providers and healthcare professionals from a variety of disciplines.

Earlier analysis of data, including qualitative data, from the wider project, suggested that attitudes and fear of infection may be the most salient drivers of HIV-related stigma in the Irish context. Both PLHIV and healthcare workers’ reports indicated that stigmatising practices were relatively common [23]. Consequently, we wanted to investigate in more depth correlates and predictors of these drivers and practices. In this study therefore, we draw on quantitative data gathered from the survey of healthcare workers. The aim of the study is to identify what individual, contextual clinic, and policy level factors play a role in (i) stigmatising practices (excessive or inappropriate infection precaution measures), (ii) stigmatising attitudes towards people living with HIV, and (iii) fear of occupational transmission among healthcare workers in the Republic of Ireland.

## Methods

### Survey

We used the brief standardised tool for measuring HIV-related stigma among health facility staff (17), adapted with input from a stakeholder panel. The wider project of which this study was a part adopted a collaborative joint-stakeholder approach; this involved the participation of a stakeholder panel that included healthcare workers (*n* = 4) and people living with HIV (*n* = 5) [21]. The stakeholder panel advised on adaptations to data collection tools, including this survey. Adaptations included slight changes to language, and sex and gender categories. The panel also advised on the inclusion of a question on knowledge of U = U. This abbreviation signifies ‘undetectable equals untransmittable’, a widely adopted shorthand descriptor to express the knowledge that effective treatment with antiretroviral therapy makes it impossible to transmit HIV. The questionnaire contained 22 core questions capturing information on socio-demographic variables, contextual clinic level variables and policy level variables, and standardised measures of stigma practices, attitudes, and fear of occupational HIV transmission. The questionnaire was distributed through the Qualtrics survey platform and took approximately 15 min to complete.

Ethical approval for the study was granted by the Research Ethics Committee at the University of Galway, and data were processed in line with European General Data Protection Regulations (GDPR). Participant information on the aims and purpose of the study was presented, and informed consent obtained from all participants before proceeding with the survey.

We aimed to recruit a diverse convenience sample of healthcare workers nationally from a range of settings and disciplines. Ireland’s healthcare system includes a complex mix of private and public providers, thus a variety of methods were necessary. These included: advertisements through multiple hospital staff apps, newsletters, and ezines; email invitations and advertising via professional organisations and trade unions; phone invitations to a sample of General Practitioners and other primary care staff from every county in the country; a social media campaign (Twitter, LinkedIn, Instagram); promotion via national media and sector-specific publications; and WhatsApp messages through informal professional networks. Recruitment materials contained a link or QR code through which participants could access the questionnaire, which was open to anyone working in the health service in the Republic of Ireland, including those in non-clinical roles.

Data were collected over nine weeks from the end of May 2022 through to the end of July 2022. The questionnaire was opened by 378 respondents, of whom 298 (78.8%) provided complete responses. Incomplete responses were excluded from analysis. Three participants were excluded because they did not provide information on their gender, length of service, and experience of working in a HIV clinic. The analytic sample therefore included 295 participants. In calculating outcome scale variables, listwise deletion was used.

### Outcomes of Interest

Stigmatising attitudes were captured with five items scored on a four-point scale reverted into strongly disagree = 0, disagree = 1, agree = 2 and strongly agree = 3. A sum score of reverted items was calculated, resulting in a scale ranging from 0 to 15, where higher scores reflect more stigmatising attitudes. The scale showed internal consistency on the verge of the acceptable threshold (Cronbach’s alpha = 0.67).

Stigmatising practices were captured by four items measuring infection precaution measures used by healthcare workers. These were dichotomised into 0 (not employing the given measure) versus 1 (employing the given measure). Those who responded ‘not applicable’ to any of the items were excluded from the analysis. A sum score of the dichotomous items was calculated, ranging from 0 to 4, where higher scores reflect more stigmatising measures. The scale had low internal consistency (Cronbach’s alpha = 0.63).

Four items capturing fear of occupational transmission of HIV scored on a five-point scale were reverted into not worried = 0, a little worried = 1, worried = 2, very worried = 3. Those who responded ‘not applicable’ to any of the items were excluded from the analysis. A sum score of reverted items was calculated, resulting in a scale ranging from 0 to 12, where higher scores reflect more fear of contracting HIV. The scale had acceptable internal consistency (Cronbach’s alpha = 0.76).

### Individual Level Predictor Measures

Individual level measures captured respondent data on age, gender, profession, length of time spent working in healthcare, ever having worked in a HIV clinic, and agreement with/knowledge of U = U. Five response options were available for the latter measure: ‘strongly agree’; ‘agree’; ‘disagree’; ‘strongly disagree’; ‘don’t know’. These were dichotomised into ‘agree and strongly agree’ versus ‘disagree, strongly disagree or don’t know’ for the purposes of analysis.

### Contextual Clinic Level Predictor Measures

Contextual clinic level measures captured data on the type of facility or healthcare service in which the respondent worked and the number of patients with HIV cared for in the previous 12 months.

### Policy Level Predictor Measures

Policy level measures captured data on training received by the healthcare worker (four items) and knowledge/existence of policies and practices (six items) in the workplace.

### Statistical Analysis

Analysis was carried out in IBM SPSS Statistical Software, Version 28 (Armonk, NY, USA). After creating descriptive statistics for all variables, we checked the bivariate associations between predictor variables and the outcome variables of stigmatising attitudes, stigmatising practices (infection precaution measures), and fear of occupational transmission of HIV. Student’s *t*-tests were used to test associations with binary variables, one-way analysis of variance (ANOVA) for categorical variables, and Pearson’s *r* for continuous variables. Due to non-normal distributions demonstrated by visual inspection of the histograms and Kolmogorov-Smirnov and Shapiro-Wilk tests (*p* < .001) we repeated the analyses using non-parametric techniques, which rendered identical results to those of their parametric equivalents. Effect size *r* values (*r*_ES_) were calculated for associations between predictor and binary outcome variables. We interpreted these using Funder and Ozer’s [24] guidelines: *r*_ES_ ≤ 0.05 very small, *r*_ES_ = 0.10 small, *r*_ES_ = 0.20 medium, *r*_ES_ = 0.30 large, and *r*_ES_ ≥ 0.40 very large effects. For associations between predictor and continuous variables we obtained random-effect omega squared (*ω*^2^) effect sizes, and interpreted them using Kirk’s [25] guidelines: *ω*^2^ ≤ 0.010 small, *ω*^2^ = 0.059 medium, *ω*^2^ ≥ 0.138 large effects.

Finally, we built three consecutive multiple linear regression models that predicted stigmatising attitudes, stigmatising practices, and fear of occupational HIV transmission. Model 1 included individual level predictors; Model 2 added contextual clinic level predictors; and Model 3 added policy level predictors. Multicollinearity was tested using inter-variable correlations (threshold: *r <* .90) and the variance inflation factor (threshold: VIF < 5). For each all-inclusive multiple regression model, we examined outliers by exploring standardised residuals and Mahalanobis distances. In line with earlier practice [26] and methodological recommendations [27], we excluded cases with a standardised residual exceeding 3, and/or Mahalanobis distance exceeding 25. Four participants were excluded from the model on stigmatising attitudes, leaving 260 participants in the final model. Four participants were excluded from the model on stigmatising practices, leaving 208 participants in the final model. Seven participants were excluded from the model on fear of occupational transmission, resulting in a final model with 193 participants.

## Results

### Sample Characteristics and Variable Distributions

Sample characteristics are presented in Table 1. A diverse sample of healthcare workers, with a wide range of age and professional experience participated in the study. The sample has a gender imbalance; almost 80% of the participants were female. This, however, is line with the general gender composition of the healthcare workers in Ireland [28]. Most respondents were aged 41–50. At 34%, nurses were in the majority, followed by allied health professionals (23.4%) and doctors (22.7%). Approximately a quarter of respondents had worked 20–30 years in the health service; 29.2% had worked previously in a HIV clinic; and a majority (65.8%) worked in a hospital setting. A majority (42.9%) reported they saw no (or were unsure if they saw) patients with HIV in the previous 12 months. A majority (76.9%) agreed or strongly agreed with the U = U statement, while 6.4% expressed disagreement or strong disagreement, and 16.6% indicated not knowing.

**Table 1:** Sample characteristics (*N* = 295)

**Variable**	***n***	**%**
**Gender**		
Male	63	21.4
Female	232	78.6
**Age**		
Under 30	69	23.4
31–40	67	22.7
41–50	90	30.5
Over 50	69	23.4
**Profession**		
Nurse	100	33.9
Doctor	67	22.7
Allied health professional	69	23.4
Dental professional	32	10.8
Management and admin	27	9.2
**Years worked in the health service**		
Less than 5 years	51	17.3
5–10 years	65	22.0
10–20 years	70	23.7
20–30 years	73	24.7
More than 30 years	36	12.2
**Ever worked in a HIV clinic**		
Yes	86	29.2
No	209	70.8
**Type of healthcare service**		
Hospital	194	65.8
Community care	40	13.6
Dental	24	8.1
Private	13	4.4
Other	16	5.4
Missing response	8	2.7
**Number of HIV patients cared for in the last 12 months**		
None or unsure	126	42.7
1 to 10	118	40.0
11 to 100	26	8.8
More than 100	16	5.4
Missing response	9	3.1
**Agreement with U = U**		
Agrees	227	76.9
Disagrees or does not know	68	23.1

Table 2 displays the distribution of the policy level variables. Most respondents received training on infection control and universal precautions (75.6%) and informed consent, privacy and confidentiality (72.5%). Much fewer received training in HIV stigma and discrimination (20.3%), or stigma and discrimination towards key populations (29.5%). Many participants responded in the affirmative for five of the policy items (64.4–92.5%), while a fifth only indicated that their facility had written guidelines to protect patients living with HIV from discrimination. The distribution of the composite scores for these variables can be seen in Table 3.

**Table 2:** Distribution of policy level variables (*N* = 295)

**Variables**						
**Training**	**Received training**		**Not received training**		**Missing**	
	***n***	**%**	***n***	**%**	***n***	**%**
Received training on HIV and discrimination	60	20.3	235	79.7	0	0
Received training on infection control and universal precautions	223	75.6	72	24.4	0	0
Received training on informed consent, privacy and confidentiality	214	72.5	81	27.5	0	0
Received training on stigma and discrimination towards specific populations	87	29.5	208	70.5	0	0
**Policies**	**Policy exists**		**No policy or not applicable**		**Missing**	
	***n***	**%**	***n***	**%**	***n***	**%**
In my facility it is not acceptable to test a patient for HIV without their knowledge.	190	64.4	102	34.6	3	1.0
I will get into trouble at work if I discriminate against patients living with HIV.	225	76.3	68	23.1	2	0.7
I have access to adequate supplies in my health facility to reduce my risk of becoming infected with HIV.	230	78.0	64	21.7	1	0.3
There are standardised procedures/protocols in my health facility to reduce my risk of becoming infected with HIV.	217	73.6	78	26.4	0	0
There are procedures/protocols in my health facility for needlestick injuries and/or other exposure incidents.	273	92.5	22	7.5	0	0
My health facility has written guidelines to protect patients living with HIV from discrimination.	59	20.0	235	79.7	1	0.3

**Table 3:** Distribution of the training composite score and the policies composite score

**Training (** ***N*** ** = 295)**			**Policies (** ***N*** ** = 292)**		
**Score**	***n***	**%**	**Score**	***n***	**%**
0	38	12.9	0	5	1.7
1	63	21.4	1	4	1.4
2	109	36.9	2	27	9.2
3	37	12.5	3	52	17.8
4	48	16.3	4	75	25.7
			5	97	33.2
			6	32	11.0

Table 4 contains the distribution of the outcome variables. Most respondents did not endorse stigmatising attitudes towards PLHIV (90–100%). In respect of stigmatising practices, few respondents reported avoiding contact (4.5%) or double gloving (15.2%). A greater proportion reported wearing gloves during all aspects of a patient’s care (35.7%) and just over a quarter reported using special measures they would not use with other patients. In respect of the fear of occupational transmission variables, very few respondents indicated they would be worried about casual contact. A greater proportion reported they would worry dressing wounds (38.5%) or drawing blood (53.1%) of a person living with HIV. Table 5 shows the composite scores for these variables.

**Table 4:** Distribution of attitudes, infection precaution measures and fear

**Stigmatising attitudes (** ***N*** ** = 280)**	**Agree**		**Disagree**	
	***n***	**%**	***n***	**%**
Most people living with HIV do not care if they infect other people.	5	1.8	275	98.2
People living with HIV should feel ashamed of themselves.	3	1.1	277	98.9
Most people living with HIV have had many sexual partners.	14	5.0	266	95.0
People get infected with HIV because they engage in irresponsible behaviours.	28	10.0	252	90.0
HIV is punishment for bad behaviour.	0	0.0	280	100.0
**Infection precaution measures (** ***N*** ** = 224)** ^a^	**Yes**		**No**	
	***n***	**%**	***n***	**%**
Avoiding contact	10	4.5	214	95.5
Wearing double gloves	34	15.2	190	84.8
Wearing gloves throughout the whole procedure	80	35.7	144	64.3
Using special measures	56	25.0	168	75.0
**Fear (** ***N*** ** = 213)** ^a^	**Worried**		**Not worried**	
	***n***	**%**	***n***	**%**
Touching clothes of a HIV patient	14	6.6	199	93.4
Dressing the wound of a HIV patient	82	38.5	131	61.5
Taking blood from a HIV patient	113	53.1	100	46.9
Taking temperature of a HIV patient	7	3.3	206	96.7

^a^Those responding ‘not applicable’ to any of the items were excluded from the analysis

**Table 5:** Distribution of the attitudes, infection precaution measures and fear scale scores

**Stigmatising attitudes** ^**a**^ **(** ***N*** ** = 280)**			**Infection precaution measures** ^**b**^ **(** ***N*** ** = 224)**			**Fear** ^**b**^ **(** ***N*** ** = 213)**		
**Score**	***n***	**%**	**Score**	***n***	**%**	**Score**	***n***	**%**
0	104	37.1	0	119	53.1	0	97	45.5
1	57	20.4	1	54	24.1	1	35	16.4
2	46	16.4	2	34	15.2	2	41	19.2
3	36	12.9	3	10	4.5	3	15	7.0
4	24	8.6	4	7	3.1	4	7	3.3
5	8	2.9				5	7	3.3
6	2	0.7				6	4	1.9
7	2	0.7				7	2	0.9
8	1	0.4				8	3	1.4
9	0	—				9	1	0.5
10	0	—				10	0	—
11	0	—				11	0	—
12	0	—				12	1	0.5
13	0	—						
14	0	—						
15	0	—						

^a^Listwise deletion was used for missing responses. ^b^Listwise deletion was used for missing and ‘not applicable’ responses

### Bivariate Results

Tables 6, 7 and 8 show bivariate associations of sample characteristics and stigmatising attitudes, behaviours and fears respectively. Only being male was identified as being associated with stigmatising attitudes, with a medium effect: *t* (74.37) = − 2.06, *p =* .043, *r*_ES_ = 0.232. Several factors were identified as being associated with stigmatising practices, including never having worked in a HIV clinic, with a large effect size: *t* (201.80) = 4.78, *p <* .001, *r*_ES_ = 0.318; being a dental professional, with a small effect size: *F* (4) = 3.82, *p =* .005, *ω*^2^ = 0.012; working in a dental setting, with a small effect size: *F* (4) = 2.88, *p =* .024, *ω*^2^ = 0.009; being unsure of how many HIV patients one has cared for, or having less than ten HIV patients in the previous 12 months, with a small effect size: *F* (3) = 3.21, *p =* .024, *ω*^2^ = 0.010; disagreeing with or not knowing about U = U, with a very large effect size: *t* (52.86) = 4.10, *p <* .001, *r*_ES_ = 0.491; and a higher composite score on policies and practices: *r =* .145; *p =* .030. In respect of fear of occupational transmission, the factors identified as having an association included: younger age, with a small effect size: *F* (3) = 6.63, *p <* .001; *ω*^2^ = 0.026; having worked for a shorter period in the health service, with a small effect size: *F* (4) = 6.03, *p <* .001, *ω*^2^ = 0.023; never having worked in a HIV clinic, with a medium effect size: *t* (170.14) = − 3.47, *p* < .001, *r*_ES_ = 0.257; and disagreeing with or not knowing about U = U, with a large effect size: *t* (56.94) = − 2.89, *p* = .006, *r*_ES_ = 0.358.

**Table 6:** Bivariate associations of sample characteristics and stigmatising attitudes

**Variable**	**M (SD)**	**Test statistics (df)**	***p*** **-value**	**Effect size**	**Comparisons**
**Gender**					
Male (*n* = 58)	1.98 (2.00)	*t* (74.37) = −2.06	0.043	*r*_ES_ = 0.232	Male > Female
Female (*n* = 222)	1.41 (1.49)				
**Age**					
Under 30 (*n* = 65)	1.58 (1.66)	*F* (3) = 0.69	0.558		
31–40 (*n* = 65)	1.57 (1.65)				
41–50 (*n* = 82)	1.32 (1.68)				
Over 50 (*n* = 68)	1.68 (1.49)				
**Profession**					
Nurse (*n* = 94)	1.71 (1.85)	*F* (4) = 0.88	0.477		
Doctor (*n* = 64)	1.52 (1.38)				
Allied health professional (*n* = 67)	1.28 (1.41)				
Dental professional (*n* = 29)	1.69 (1.58)				
Management and admin (*n* = 26)	1.31 (1.81)				
**Years worked in the health service**					
Less than 5 years (*n* = 48)	1.44 (1.69)	*F* (4) = 0.84	0.502		
5–10 years (*n* = 64)	1.72 (1.56)				
10–20 years (*n* = 62)	1.48 (1.73)				
20–30 years (*n* = 71)	1.31 (1.50)				
More than 30 years (*n* = 35)	1.80 (1.68)				
**Ever worked in a HIV clinic**					
Yes (*n* = 80)	1.29 (1.37)	*t* (179.53) = −1.71	0.090		
No (*n* = 200)	1.62 (1.70)				
**Type of healthcare service**					
Hospital (*n* = 183)	1.55 (1.62)	*F* (4) = 0.97	0.426		
Community care (*n* = 38)	1.34 (1.53)				
Dental (*n* = 24)	1.79 (1.58)				
Private (*n* = 11)	1.55 (1.57)				
Other (*n =* 16)	0.88 (1.15)				
**Number of HIV patients cared for in the last 12 months**					
None or unsure (*n* = 120)	1.53 (1.64)	*F* (3) = 0.79	0.501		
1 to 10 (*n* = 111)	1.64 (1.73)				
11 to 100 (*n* = 25)	1.12 (1.20)				
More than 100 (*n* = 16)	1.31 (1.52)				
**Agreement with U = U**					
Agrees (*n* = 217)	1.43 (1.60)	*t* (278) = −1.77	0.078		
Disagrees or does not know (*n* = 63)	1.84 (1.67)				
**Training**	1.97 (1.22)	*r =* −.063	0.296		
**Policies and practices**	4.08 (1.29)	*r* = −.106	0.080		

*Note* Student’s *t*-tests were conducted for dichotomous variables; between-subjects analyses of variance for categorical variables, and Pearson’s *r* for continuous variables. *r*_ES_ = effect size *r*

**Table 7:** Bivariate associations of sample characteristics and stigmatising practices (infection precaution measures)

**Variable**	**M (SD)**	**Test statistics (df)**	***p*** **-value**	**Effect size**	**Comparisons**
**Gender**					
Male (*n* = 46)	0.96 (1.28)	*t* (222) = −1.11	0.270		
Female (*n* = 178)	0.76 (0.99)				
**Age**					
Under 30 (*n* = 54)	0.98 (1.27)	*F* (3) = 1.09	0.355		
31–40 (*n* = 56)	0.88 (1.08)				
41–50 (*n* = 70)	0.69 (1.00)				
Over 50 (*n* = 44)	0.68 (0.77)				
**Profession**					
Nurse (*n* = 94)	0.82 (1.06)	*F* (4) = 3.82	0.005	*ω*^2^ = 0.012	Dental professional > Allied health professional
Doctor (*n* = 57)	0.65 (1.01)				
Allied health professional (*n* = 39)	0.51 (0.82)				
Dental professional (*n* = 29)	1.28 (1.00)				
Management and admin (*n* = 5)	1.80 (2.05)				
**Years worked in the health service**					
Less than 5 years (*n* = 33)	0.94 (1.35)	*F* (4) = 0.66	0.622		
5–10 years (*n* = 53)	0.85 (1.08)				
10–20 years (*n* = 51)	0.86 (1.15)				
20–30 years (*n* = 61)	0.62 (0.86)				
More than 30 years (*n* = 26)	0.85 (0.78)				
**Ever worked in a HIV clinic**					
Yes (*n* = 73)	0.40 (0.74)	*t* (201.80) = 4.78	< 0.001	*r*_ES_ = 0.318	No > Yes
No (*n* = 151)	1.00 (1.13)				
**Type of healthcare service**					
Hospital (*n* = 149)	0.78 (1.10)	*F* (4) = 2.88	0.024	*ω*^2^ = 0.009	Dental > Community care, Other setting
Community care (*n* = 27)	0.63 (0.79)				
Dental (*n* = 22)	1.41 (1.05)				
Private (*n* = 11)	0.55 (0.69)				
Other (*n =* 8)	0.25 (0.46)				
**Number of HIV patients cared for in the last 12 months**					
None or unsure (*n* = 79)	0.90 (1.06)	*F* (3) = 3.21	0.024	*ω*^2^ = 0.010	None or unsure, 1 to 10 > More than 100
1 to 10 (*n* = 103)	0.90 (1.11)				
11 to 100 (*n* = 23)	0.52 (0.99)				
More than 100 (*n* = 11)	0.00 (0.00)				
**Agreement with U = U**					
Agrees (*n* = 181)	0.64 (0.92)	*t* (52.86) = 4.10	< 0.001	*r*_ES_ = 0.491	Disagrees or does not know > Agrees
Disagrees or does not know (*n* = 43)	1.49 (1.28)				
**Training**	1.98 (1.22)	*r* = −.093	0.166		
**Policies and practices**	4.08 (1.30)	*r* = .145	0.030		

*Note* Student’s *t*-tests were conducted for dichotomous variables; between-subjects analyses of variance were for categorical variables; and Pearson’s *r* for continuous variables. *r*_ES_ = effect size *r*. *ω*^2^ = omega-squared effect size (random effects)

**Table 8:** Bivariate associations of sample characteristics and fear of occupational transmission

**Variable**	**M (SD)**	**Test statistics (df)**	***p*** **-value**	**Effect size**	**Comparisons**
**Gender**					
Male (*n* = 50)	1.38 (2.20)	*t* (211) = 0.27	0.789		
Female (*n* = 163)	1.47 (1.92)				
**Age**					
Under 30 (*n* = 54)	2.22 (2.14)	*F* (3) = 6.63	< 0.001	*ω*^2^ = 0.026	Younger than 30 > 41–50, older than 50
31–40 (*n* = 46)	1.85 (2.27)				
41–50 (*n* = 67)	0.96 (1.81)				
Over 50 (*n* = 46)	0.85 (1.25)				
**Profession**					
Nurse (*n* = 94)	1.37 (2.09)	*F* (4) = 3.49	0.009	*ω*^2^ = 0.012	No significant differences in pairwise comparisons
Doctor (*n* = 62)	1.02 (1.40)				
Allied health professional (*n* = 25)	2.36 (2.36)				
Dental professional (*n* = 17)	1.12 (1.62)				
Management and admin (*n* = 15)	2.53 (2.42)				
**Years worked in the health service**					
Less than 5 years (*n* = 35)	2.29 (2.44)	*F* (4) = 6.03	< 0.001	*ω*^2^ = 0.023	Less than 5 years, 5–10 years, 10–20 years > 20–30 years
5–10 years (*n* = 47)	1.83 (1.92)				
10–20 years (*n* = 52)	1.71 (2.47)				
20–30 years (*n* = 54)	0.52 (0.80)				
More than 30 years (*n* = 25)	1.00 (1.16)				
**Ever worked in a HIV clinic**					
Yes (*n* = 67)	0.84 (1.54)	*t* (170.14) = −3.47	< 0.001	*r*_ES_ = 0.257	No > Yes
No (*n* = 146)	1.73 (2.10)				
**Type of healthcare service**					
Hospital (*n* = 152)	1.42 (1.98)	*F* (4) = 0.52	0.720		
Community care (*n* = 26)	1.58 (2.02)				
Dental (*n* = 11)	1.55 (1.81)				
Private (*n* = 6)	0.33 (0.82)				
Other (*n =* 10)	1.60 (2.50)				
**Number of HIV patients cared for in the last 12 months**					
None or unsure (*n* = 84)	1.64 (1.97)	*F* (3) = 1.80	0.148		
1 to 10 (*n* = 89)	1.40 (2.16)				
11 to 100 (*n* = 22)	1.09 (1.51)				
More than 100 (*n* = 11)	0.27 (0.65)				
**Agreement with U = U**					
Agrees (*n* = 168)	1.21 (1.77)	*t* (56.94) = −2.89	0.006	*r*_ES_ = 0.358	Disagrees or does not know > Agrees
Disagrees or does not know (*n* = 45)	2.33 (2.45)				
**Training**	1.97 (1.21)	*r* = −.097	0.159		
**Policies and practices**	4.17 (1.28)	*r* = −.126	0.068		

*Note* Student’s *t*-tests were conducted for dichotomous variables; between-subjects analyses of variance were for categorical variables; and Pearson’s *r* for continuous variables. *r*_ES_ = effect size *r*. *ω*^2^ = omega-squared effect size (random effects)

### Multivariate Results

The results of the multiple regression are displayed in Tables 9, 10 and 11 for analysis of stigmatising attitudes, stigmatising practices, and fear of occupational transmission respectively. Model 1 tested individual level predictors: gender, age, profession, years worked in healthcare, ever worked in a HIV clinic and knowledge of U = U. Model 2 added in contextual clinic predictors: type of healthcare service and number of HIV patients seen in the last 12 months. Model 3 added in policy level predictors: training received and institutional policies.

**Table 9:** Multiple regression analyses predicting stigmatising attitudes toward patients living with HIV (*N* = 260)

	**Model 1**			**Model 2**			**Model 3**			
	**B**	**95% CI (B)**	***β***	**B**	**95% CI (B)**	***β***	**B**	**95% CI (B)**		***β***
**Constant**	2.47^***^	1.69; 3.24		2.49^***^	1.62; 3.36		2.90^***^	1.90; 3.90		
**Predictor variables**										
Gender	−0.36	–0.81; 0.10	−0.10	–0.35	–0.81; 0.10	−0.10	–0.35	–0.81; 0.10		–0.10
Age	0.04	–0.25; 0.33	0.03	0.03	–0.27; 0.33	0.02	0.04	–0.26; 0.34		0.03
Profession	**−0.15** ^*^	**–0.30; − 0.01**	**−0.13**	−0.14	–0.30; 0.02	−0.12	**–0.16** ^*^	**–0.32; 0.01**		**−0.14**
Years worked in the health service	−0.04	–0.29; 0.22	−0.03	–0.03	–0.29; 0.23	−0.02	**–**0.003	–0.27; 0.26		**–**0.002
Ever worked in a HIV clinic	–0.26	−0.68; 0.16	–0.08	–0.27	–0.72; 0.18	−0.08	–0.26	–0.71; 0.20		–0.08
Knowledge of U = U	–0.39	−0.84; 0.06	–0.11	–0.38	–0.84; 0.08	–0.11	–0.37	–0.84; 0.09		–0.10
Type of healthcare service				–0.04	−0.21; 0.13	–0.03	–0.03	–0.20; 0.14		–0.03
Number of HIV patients in the last 12 months				0.001	–0.24; 0.24	0.001	0.03	–0.21; 0.27		0.02
Training							–0.001	–0.16; 0.16		0.00
Institutional policies							–0.13	–0.29; 0.03		−0.11
**Model fit statistics**										
*R*^2^	0.043			0.044			0.054			
*F*	1.90 (*ns.*)			1.45 (*ns.*)			1.43 (*ns.*)			
Δ*R*^2^				0.001 (*ns.*)			0.010 (*ns.*)			

^*^p < .05; ^**^p < .01; ^***^p < .001. For the model fit statistics, we also indicated non-significant results (*ns.*) Significant predictors are highlighted in bold letters

**Table 10:** Multiple regression analyses predicting stigmatising practices toward patients living with HIV (*N* = 208)

	**Model 1**			**Model 2**			**Model 3**			
	**B**	**95% CI (B)**	***β***	**B**	**95% CI (B)**	***β***	**B**		**95% CI (B)**	***Β***
**Constant**	1.68^***^	1.14; 2.24		1.71^***^	1.11; 2.31		1.00^**^		0.26; 1.73	
**Predictor variables**										
Gender	–0.10	–0.43; 0.23	–0.04	–0.10	–0.43; 0.24	–0.04	–0.04		–0.37; 0.28	–0.02
Age	–0.14	–0.39; 0.12	–0.14	–0.14	–0.39; 0.12	–0.14	–0.09		–0.34; 0.16	–0.09
Profession	0.05	–0.07; 0.16	0.05	0.04	–0.09; 0.17	0.05	0.09		–0.04; 0.22	0.10
Years worked in the health service	0.10	–0.12; 0.31	0.12	0.09	–0.13; 0.31	0.12	0.01		–0.20; 0.23	0.02
Ever worked in a HIV clinic	**–0.45** ^***^	**–0.73; − 0.18**	**–0.21**	**–0.44** ^**^	**–0.74; − 0.15**	**–0.21**	**–0.43** ^**^		**–0.73; − 0.13**	**–0.20**
Knowledge of U = U	**–0.91** ^***^	**–1.24; − 0.57**	**–0.35**	**–0.90** ^***^	**–1.24; − 0.56**	**–0.35**	**–0.84** ^***^		**–1.18; − 0.51**	**–0.33**
Type of healthcare service				0.01	–0.13; 0.15	0.01	0.005	–0.13; 0.14		0.005
Number of HIV patients in the last 12 months				–0.01	–0.19; 0.16	–0.01	–0.04	–0.21; 0.14		–0.03
Training							–0.08	–0.19; 0.03		0.10
Institutional policies							**0.21** ^**^	**0.09; 0.32**		**0.24**
**Model fit statistics**										
*R*^2^	0.205			0.205			0.253			
*F*	8.61^***^			6.41^***^			6.67^***^			
Δ*R*^2^				< 0.001 (*ns.*)			0.05^**^			

^*^p < .05; ^**^p < .01; ^***^p < .001. For the model fit statistics, we also indicated non-significant results (*ns.*) Significant predictors are highlighted in bold letters

**Table 11:** Multiple regression analyses predicting fear of occupational transmission of HIV (*N* = 193)

	**Model 1**			**Model 2**			**Model 3**		
	**B**	**95% CI (B)**	***β***	**B**	**95% CI (B)**	***β***	**B**	**95% CI (B)**	***β***
**Constant**	2.77^***^	1.87; 3.67		3.15^***^	2.12; 4.17		3.38^***^	2.16; 4.60	
**Predictor variables**									
Gender	**0.58** ^*^	**0.05; 1.11**	**0.14** ^*^	**0.60** ^*^	**0.07; 1.13**	**0.15** ^*^	**0.62** ^*^	**0.09; 1.16**	**0.15** ^*^
Age	−0.35	−0.77; 0.07	−0.22	−0.38	−0.80; 0.04	–0.24	−0.37	–0.80; 0.05	−0.24
Profession	0.12	−0.07; 0.31	0.08	0.11	−0.09; 0.31	0.07	0.09	−0.11; 0.30	0.06
Years worked in the health service	–0.14	–0.51; 0.23	–0.10	−0.12	−0.50; 0.25	–0.09	−0.12	−0.50; 0.27	–0.09
Ever worked in a HIV clinic	**–0.61** ^*^	**−1.10; −0.12**	**–0.17** ^*^	−0.44	–0.98; 0.10	–0.12	−0.38	–0.94; 0.18	–0.10
Knowledge of U = U	–0.88**	**–1.45; − 0.30**	**− 0.21**	–0.84**	**–1.42; − 0.27**	**–0.20**	**−0.85** ^**^	**−1.43; − 0.27**	**–0.20** ^**^
Type of healthcare service				−0.02	–0.23; 0.19	−0.01	**−**0.004	−0.22; 0.21	**−0.002**
Number of HIV patients in the last 12 months				−0.23	–0.52; 0.07	–0.11	−0.22	–0.52; 0.08	−0.11
Training							–0.09	−0.29; 0.11	–0.06
Institutional policies							−0.04	−0.24; 0.17	–0.03
**Model fit statistics**									
*R*^2^	0.224			0.234			0.238		
*F*	8.95^***^			7.01^***^			5.70^***^		
Δ*R*^2^				0.010 (*ns.*)			0.005 (*ns.*)		

^*^p < .05; ^**^p < .01; ^***^p < .001. For the model fit statistics, we also indicated non-significant results (*ns.*) Significant predictors are highlighted in bold letters

Albeit profession had significant contribution to stigmatising attitudes in Model 1 and Model 3, neither the other predictors, nor the models themselves were significant. Ever having worked at a HIV clinic, and knowledge of U = U were significant predictors of stigmatising practices in all three models; in the third model, ever having worked at a HIV clinic, agreement with U = U, and institutional policies showed significant contribution. These three factors explained 25.3% variance in total. In respect of fear of occupational transmission of HIV, gender, ever having worked at a HIV clinic and knowledge of U = U were significant predictors in the first model, but only gender and knowledge of U = U retained their explanative power in the second and the third model, accounting for 23.8% of variance in total. For both stigmatising practices and fear of occupational transmission, knowledge of U = U was the most powerful predictor.

## Discussion

In this paper we have reported findings from the first quantitative study of HIV-related stigma among healthcare workers in the Republic of Ireland. To our knowledge, this is also the first study of HIV stigma among healthcare workers anywhere to include an indicator on knowledge of U = U. The findings suggest knowledge of U = U is the most powerful protective factor against stigmatising practices and fear of occupational transmission of HIV in healthcare settings. Several other individual level, contextual clinic, and policy level factors were also found to be significant predictors.

### The Findings in International Context

An encouraging finding is that stigmatising attitudes towards people living with HIV in the Republic of Ireland were relatively rare. This aligns with results from another European study [29], which similarly reported lower stigmatising attitudes than have been found in the US [26, 30], Iran [31] and other non-European countries [17]. While stigmatising attitudes were generally rare, bivariate analysis suggested that male healthcare workers had more stigmatising attitudes towards people living with HIV. This corresponds with similar findings from the US [26, 32] and Kazakhstan [33], but is contrary to findings from Laos [34], Saudi Arabia [35] and India [36]. This divergence would suggest, as per the Health Stigma and Discrimination Framework, that stigmatising attitudes depend to some extent on socio-cultural context. Despite the medium effect size of the association between male gender and stigmatising attitudes apparent in the bivariate analysis, and a very small effect of profession in the multiple regression, the model was not statistically significant.

Fear of occupational transmission of HIV among healthcare workers in this study was prevalent, with 38.5% of HCWs working in clinical settings reporting worry about dressing a wound of PLHIV and 53.1% reporting worry about drawing blood. Still, these figures compare somewhat favourably to findings from a recent study in the Netherlands, where 73% expressed worry about drawing blood and 65% dressing a wound [37]. The findings nevertheless suggest that fear of infection rather than social prejudice is driving stigmatising practices among healthcare workers in the Irish context [38]. Such stigmatising practices were not uncommon; a quarter of healthcare workers reported using special measures with patients living with HIV. 36% also reported wearing gloves throughout all aspects of a patient’s care. This latter finding should, however, be interpreted with caution, given the increased use of personal protective equipment as a result of the COVID-19 pandemic.

Stigmatising practices were positively predicted by lack of knowledge of U = U, never having worked in a HIV clinic previously and a higher score on the institutional policies scale. Bivariate analysis indicated that excessive use of infection precaution measures was associated with healthcare workers in the dental profession; those with no experience working in a HIV clinic; those who worked in a hospital or dental setting; and lack of knowledge of U = U. High levels of stigma among dentists and lower stigma among those who had worked previously with PLHIV have been reported previously in Iran [31]. Fear of occupational transmission was positively predicted by female gender and a lack of knowledge of U = U. Specific factors identified as being associated with fear of occupational transmission included younger age, having less experience working in healthcare, and never having worked in a HIV clinic, all of which have been identified previously elsewhere as salient factors in stigma among healthcare workers [34, 39–41].

### Interpreting the Findings and Implications for Future Research

In the Irish context, where men who have sex with men comprise the population most affected by HIV, the findings on attitudes towards people living with HIV may potentially be explained by greater social liberalisation in recent years; for instance a referendum on marriage equality passed with an overwhelming majority in 2015, suggesting greater acceptance of gay relationships [42]. At the same time, due to the sensitive topic, it is also possible that a degree of social desirability bias was a factor [43].

An additional consideration is how this finding might be interpreted with an intersectional lens [44], given the attitudes indicator questions generalise rather than specify key populations or minority groups. Certainly evidence from previous studies carried out in Ireland suggest that people living with HIV who use drugs may receive less favourable treatment than others [7], while there is evidence more broadly that ethnic minority groups, such as Travellers, are significantly more likely than the general population to experience discrimination accessing and using healthcare services [45]. While beyond the scope of this investigation, future studies may wish to explore whether intersectional stigma mediates attitudes towards people living with HIV.

A counterintuitive finding was the positive relationship between higher scores on the policy scale (i.e., having more protective policies) and engaging in stigmatising practices. Although we anticipated the opposite to be true, there are examples of similar contradictory findings in the literature. For instance, a study in India found that endorsement of universal precautions was associated with higher intention to discriminate in both and high and low risk situations [46]. Similarly, a study in Kazakhstan reported some facility policies inadvertently reinforced stigma in certain contexts [47]. While the precise dynamic at work to produce this contradictory effect is unclear, such unintended consequences of policy implementation can arise for various reasons, including poor design, bad communication and lack of consultation or evaluation [48]. Further research may be warranted to further explore this issue.

An additional area worthy of further investigation is the association of stigmatising practices with being a dental professional or working in a dental setting. Earlier analysis of qualitative data in this research project also suggested that use of excessive infection precaution measures was wide-spread among dental health professionals in Ireland [23], whilst people living with HIV have also reported stigmatising and discriminatory experiences [7]. Advocates in Ireland have consistently highlighted issues of stigmatising behaviours among dental professionals specifically. Indeed, the Irish Human Rights and Equality Commission successfully took a case recently on behalf of a woman living with HIV who had been discriminated against by a dental clinic [49]. While it is important that such legal remedies are available, the findings here indicate that dental professionals in particular may benefit from targeted training. Formative research to gain better understanding of the needs of dentists and dental hygienists may be helpful to inform intervention programmes, and the development of sector-specific guidelines to counteract stigma in dental healthcare settings.

### U = U and Implications for Intervention

A notable finding in this study was that knowledge of U = U was the most important protective factor against stigmatising practices and fear of occupational transmission. These findings have considerable implications for stigma intervention efforts. Although U = U applies to sexual transmission [2], it has been strongly suggested that the principle holds true in respect of occupational transmission [50]. While occupational exposure can be extremely anxiety-inducing for healthcare workers, knowledge of U = U, coupled with appropriate implementation of universal precautions and programmes of post-exposure prophylaxis should help ease healthcare worker anxieties and reduce the prevalence of stigma behaviours. Targeted, evidence-based messages around U = U may be effective [51] as a relatively low-cost and scalable stigma intervention. Recent endorsements of U = U from prominent organisations, including the World Health Organization [52], should help to further legitimise this message among healthcare workers.

### Limitations and Strengths

Given the methods of recruitment, it is not possible to say what the overall response rate was for this study. Owing to concerns around privacy, and in order to ensure compliance with GDPR, data were not collected on certain demographic variables, including geographic location, religion or ethnicity, which may have facilitated identification of specific healthcare workers. However, this limits the scope for performing analysis on differences between rural and urban settings or the impact of religious or cultural beliefs. The stigmatising attitudes and practices scales had lower than desirable internal consistency; this might be the consequence of the small number of items in the scales. This study was conducted on a small sample of healthcare workers in the Republic of Ireland, and findings may not be generalisable to other countries. To the best of our knowledge, however, this is the first study to measure knowledge of U = U among healthcare workers and thus provides tentative new evidence for a powerful potential counterforce against HIV stigma.

## Conclusions

In this study we have investigated factors implicated in stigmatising practices, stigmatising attitudes and fear of occupational transmission in Irish healthcare settings. While some individual, contextual and policy level factors were found to have a role in the fear of occupational transmission and in stigmatising practices, knowledge of U = U emerged as the most important protective factor against these. We encourage other researchers to include this measure in comparable future studies for replication. Policy- and decision-makers, and others involved in health systems management, need to take steps to educate healthcare workers on U = U. While no single intervention alone can be a panacea for a problem as complex as stigma, our findings nevertheless suggest that greater awareness of and acceptance of the U = U message may have a considerable impact in reducing HIV-related stigma in healthcare settings.
